# Experimental Determination of the Fluorescence Quantum Yield of Semiconductor Nanocrystals

**DOI:** 10.3390/ma4071182

**Published:** 2011-06-30

**Authors:** Julien Laverdant, Willy Daney de Marcillac, Carlos Barthou, Vu Duc Chinh, Catherine Schwob, Laurent Coolen, Paul Benalloul, Pham Thu Nga, Agnès Maître

**Affiliations:** 1 Université Pierre et Marie Curie-Paris 6, UMR 7588, INSP, 4 place Jussieu, PARIS cedex 05, France; E-Mails: julien.laverdant@insp.jussieu.fr (J.L.); willy.marcillac@insp.jussieu.fr (W.D.M.); carlos.barthou@insp.jussieu.fr (C.B.); catherine.schwob@insp.jussieu.fr (C.S.); paul.benalloul@insp.jussieu.fr (P.B.); agnes.maitre@insp.jussieu.fr (A.M.); 2 CNRS, UMR7588, INSP, Paris cedex 05, France; 3 Institute of Materials Science, Vietnam Academy of Science and Technology, 18 Hoang Quoc Viet Str., Cau giay Dist. Hanoi 71000, Vietnam; E-Mails: chinhvd@ims.vast.ac.vn (V.D.C.); ngalamvn@yahoo.com (P.T.N.)

**Keywords:** colloidal nanocrystals, fluorescence, quantum yield

## Abstract

Many studies have considered the luminescence of colloidal II–VI nanocrystals, both in solution at a collective scale and at an individual scale by confocal microscopy. The quantum yield is an important figure of merit for the optical quality of a fluorophore. We detail here a simple method to determine the quantum yield of nanocrystals in solution as a function of the absorption. For this purpose, we choose rhodamine 101 as a reference dye to measure the nanocrystal fluorescence quantum yield. The influence of the concentration on quantum yield is therefore studied for both the reference and the solutions of nanocrystals and is found to be critical for the acuity of the method. Different types of nanocrystals are studied to illustrate different quantum yield evolutions with the concentration.

## 1. Introduction

Thanks to the progress in wet chemistry, highly luminescent semiconductor nanocrystals have been achieved [1,2]. One of their most interesting properties lies in the three dimensional carrier confinement effects. By varying their size, the emission wavelength can be tuned in the visible and the near-infrared spectrum. Moreover, nanocrystals are stable and bright at room temperature and present weak photo-bleaching. These specific properties are promising for applications such as biology imaging [2] or quantum optics [3,4].

Microscopy studies on individual nanocrystals have shown that each nanocrystal switches randomly from an “on” emitting state to an “off” non-emitting state [5]. In order to reduce this blinking by protecting the nanocrystals from the influence of their environment, several groups have synthesized nanocrystals with a thicker CdS or ZnSe shell or CdS/ZnS multishells and reported a strong reduction of blinking [6,7] and better photostability [8]. Present research at the Institute of Materials Science (Hanoi) is considering the synthesis of double-shell CdSe/ZnSe/ZnS nanocrystals. In such structures, both the electron and hole are confined in the CdSe core (type I nanocrystal), while in CdSe/CdS nanocrystals the electron is delocalized in the CdS shell (type II nanocrystals) [6].

An important practical figure of merit in the context of nanocrystal synthesis optimization is their fluorescence quantum yield [9,10,11]. The quantum yield of a fluorophore is defined as the ratio of the number of emitted photons divided by the number of absorbed photons. Different strategies have been investigated to measure this quantity, depending on whether the fluorophore is in solution or in solid phase. Chemically synthesized nanocrystals are in colloidal suspension, usually with small solution concentrations (typically micromolar).

Quantum yield measurement methods in solution were reviewed in 1970 by Demas and Crosby [12]. Later improvements were afforded by instrumental developments. The absorption of a fluorophore solution is routinely obtained by passing a beam through the solution and measuring the input and output powers (assuming there is no scattering).

In order to obtain the quantum yield, one must also determine the emitted intensity (see [12] and the references therein). The difficulty is then to determine the total emission in the full solid angle. One possibility is to use an integrating sphere; another is to use the system geometry to estimate the detector collection efficiency. Alternatively, some authors have used temperature measurements (either by calorimetry [12,13,14], or by thermal lens effect [15,16]) in order to obtain the energy dissipated as heat in the solution; the emitted intensity is then obtained by subtracting the dissipated intensity from the absorbed intensity. All of these methods require special care and involve important potential errors for non-expert laboratories.

Once the quantum yield of a specific fluorophore has been determined with sufficient care, this fluorophore can be used as a reference. By comparison with the optical absorption and emission of the reference, the quantum yield of other fluorophores can be measured with more ease and a precision of a few percent. In [17], standard conditions for such measurements are defined and standard reference dyes are proposed.

The precise realization of the latter method usually involves two apparatuses. In the first system, a beam is passed through a cuvette of fluorophore solution and its transmission is compared with the transmission of a beam passing through a cuvette of the solvent alone. In the second system, the fluorophore solution is excited and the emitted light is detected at ninety degrees. In these experiments, the detected fluorescence intensity depends on the lateral position (*z*) of the detector (scaling as $e^{-\alpha z}$, where *α* is the absorption coefficient). Therefore in order to be able to compare the fluorescence from the reference and the sample of interest, the absorption coefficient must be the same for the two samples. This can be adjusted through the choice of the reference sample, the excitation wavelength or the concentration. It can however be an important source of experimental error if *α* is large. Other experimental difficulties include the reproducibility of the cuvette and detector positioning.

The role of the fluorophore concentration is also crucial, as it may lead to problems such as reabsorption of the emitted light [15] or quenching by aggregates of dye molecules [13,16,17,18]. For the case of colloidal nanocrystals, [19] has given a detailed list of experimental pitfalls and shown that, at low concentrations, ligand desorption may lead to a strong quantum yield decrease.

In this paper, we report a method for determining the quantum yield of colloidal nanocrystals by comparison with a reference dye. In order to minimize the experimental errors introduced by detector positioning, we use a single detector for transmission and fluorescence measurement. A detailed description of the experimental setup is presented. We discuss the experimental configuration which has been chosen. The reference dye, rhodamine 101, is studied as a function of concentration in order to justify the experimental conditions. The quantum yield of various nanocrystals is then obtained. We consider state-of-the-art commercial nanocrystals, and double-shell nanocrystals synthesized in the context of our ongoing effort to improve photostability.

## 2. Principle of the Quantum Yield Measurement

In our protocol, the transmission of the excitation and the fluorescence of a fluorophore solution are measured in a single spectrum, in the same direction as the excitation beam. As the detected emission is integrated over the whole cuvette, the position of the detector is less critical and it is not necessary to maintain exactly the same absorption coefficient for the fluorophore and the reference. The transmission intensity $I_{T}$ and the fluorescence intensity $I_{F}$ are obtained respectively as the integral of the spectrum over the excitation and emission wavelength intervals. These quantities are compared with the transmission spectrum measured on the cuvette filled with the solvent alone (transmission intensity $I_{0}$), under the same conditions (detection position, acquisition time, spectrometer parameters). The fluorophore absorption intensity is then $I_{0}-I_{T}$. The same steps are performed for a reference standard dye of known quantum yield $Y^{s}$, and the unknown quantum yield $Y^{x}$ is given by :

(1) $$Y^{x}=\frac{I_{F}^{x}}{I_{F}^{s}}\frac{I_{0}^{s}-I_{T}^{s}}{I_{0}^{x}-I_{T}^{x}}\frac{n_{x}^{2}}{n_{s}^{2}}Y^{s}$$

where *s* and *x* refer to the standard reference and the sample of interest respectively.

**Figure 1:**
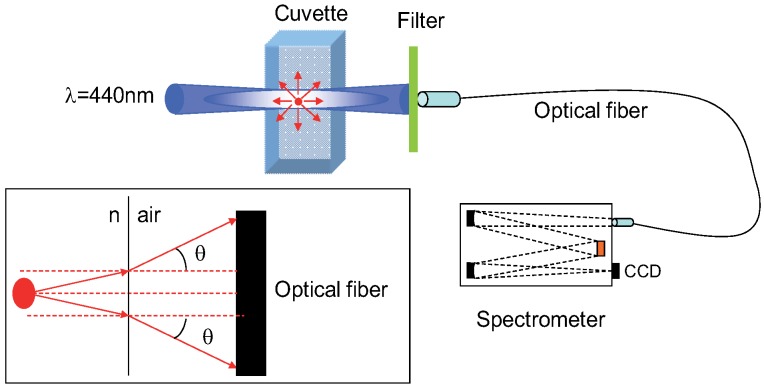
Experimental set-up for the determination of the luminescence quantum yield. The inset represents the collected solid angle and emphasizes the role of the solvent index.

Let us note that this equation contains also a term which accounts for the difference of refractive index *n* between the solvents used for the reference and the sample [12], because the solid angle which is collected by the detector scales as $n^{2}$ (see inset of Figure 1). In the case of the present work, the nanocrystals are diluted in decane ($n^{x}$ = 1.41) or toluene ($n^{x}$ = 1.49), while the standard is diluted in ethanol ($n^{s}$ = 1.36). Forgetting the refractive index term would thus lead respectively to a relative 7% or 20% underestimation of the quantum yield.

Depending on the properties of the fluorophore under consideration, different references can be chosen. Each dye exhibits a specific dependence on the emission wavelength, the pH sensitivity, the temperature and the concentration. Eaton *et al.* discussed this issue in [17] and proposed standardized references for various conditions. We choose to excite our nanocrystals at 440 nm, sufficiently far from the emission spectra (between 500 and 680 nm). Several references are available in these ranges. We choose rhodamine 101 for its high quantum yield constant with temperature, contrary to rhodamine B or 6G [20] (this behaviour is caused by the rigid structure of rhodamine 101: no rotational degree of freedom is excited [20]). We consider that the quantum yield is not dependent on the excitation wavelength, as was evidenced in [19] for rhodamine 101 in the range 490–530 nm, and for coumarin 153 over its whole excitation spectrum. The quantum yield of rhodamine 101 can however be different for a different solvent [21]. We use here ethanol, in which rhodamine has a quantum yield of 0.96 [19,20,22].

## 3. Experimental Set-up

The experimental set-up is schematized in Figure 1.

The excitation wavelength (440 nm) is selected (with a 10 nm bandwidth) from a 450 W Xenon lamp and a monochromator (Fluorolog-3). The beam is slightly focused (with an 8° angle) on a 10 mm quartz cuvette (Hellma, 111QS). In this report, all the experiments are done at room temperature.

This experimental setup provides in a single spectrum both the transmission and the emission from the samples. Therefore, errors on the position of the cuvette, the fiber and the filter are less critical. The transmitted and emitted lights from the sample are collected by an optical fiber located at 40 mm from the cuvette, on the opposite side of the excitation, with a 3 mm diameter. The collected angle is then 4 × 10${}^{-3}$. The collected light is analyzed with a Jobin-Yvon Spectrometer HR460 and a nitrogen-cooled Si Charged-Coupled Device (CCD) detector [23].

**Figure 2:**
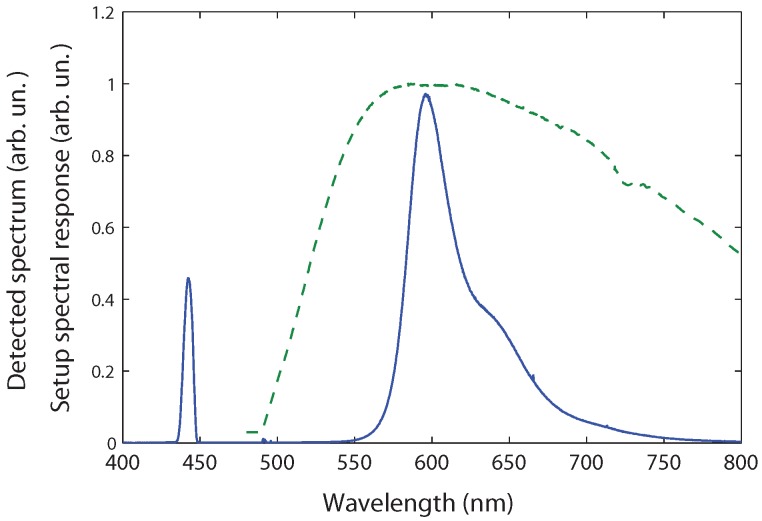
Typical measured spectrum (uncorrected) for a rhodamine 101 sample (full line) and system spectral response (dotted line), both in arbitrary units.

A typical spectrum for a rhodamine 101 solution is presented in Figure 2. The peak at 440 nm corresponds to the transmitted light through the cuvette whereas the part of the spectrum centred at 600 nm is the fluorescence of the rhodamine 101. Assuming isotropic emission, about 0.02% of the emission is collected by our fiber. On the other hand, about 2% of the transmitted beam is collected. The latter component is thus much more intense. In order to be able to measure both components in the same acquisition, we have introduced a filter which strongly decreases the transmitted component while preserving the whole emission component. For the experiments presented in this paper, a high pass filter with a cut-off at 520 nm (KV520 Schott filter) is used. Its transmission at 440 nm is 3 × 10${}^{-5}$.

When the emission spectrum is broad, or when the reference emission is not exactly at the same wavelength as the sample emission, the spectral response of the system must be taken into account. It includes the effects of the KV520 filter, the optical fiber, the monochromator and the CCD detector. We determined the spectral response of our setup by using a calibrated tungsten lamp (Figure 2). For calculating the integrated emission intensity $I_{F}$, we will always use corrected emission spectra.

## 4. Emission and Absorption Properties of Rhodamine 101

Let us first consider the measurements on the reference rhodamine 101. In order to evaluate our procedure, we have measured the variation of the ratio of the emitted to absorbed intensities for different rhodamine 101 samples with different concentrations.

**Figure 3:**
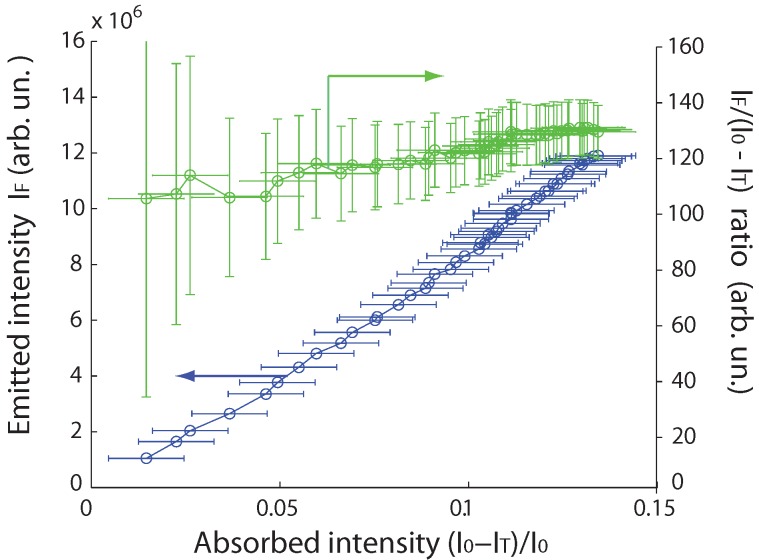
Fluorescence intensity $I_{F}$, in arbitrary units, (blue line) and ratio $I_{F}/\left(I_{0}-I_{T}\right)$ (green line) of rhodamine 101 samples (each with a different concentration) as a function of their relative absorption intensity $\left(I_{0}-I_{T}\right)/I_{0}$.

The absorption intensity $I_{0}-I_{T}$ increases with the concentration. We plot on Figure 3 the fluorescence intensity $I_{F}$ as a function of the relative absorption intensity $\left(I_{0}-I_{T}\right)/I_{0}$, with 440 nm excitation wavelength. We estimate the relative error on $I_{F}$ to $\Delta \left(I_{F}\right)/I_{F}$ = 0.5% and the error on $\left(I_{0}-I_{T}\right)/I_{0}$ to $\Delta (\left(I_{0}-I_{T}\right)/I_{0})$ = 1%, corresponding to the noise in the spectrum. By an appropriate choice of the concentration, we keep the relative absorption $\left(I_{0}-I_{T}\right)/I_{0}$ below 0.2 [24], as generally recommended by the literature [9,10,11] in order to avoid reabsorption effects. We checked (data not shown here) that the shape and position of the rhodamine 101 emission spectrum was the same for all concentrations: we conclude that neither aggregation or reabsorption effects are present in our study, which would impact the measured quantum yield [19].

The fluorescence intensity increases almost linearly as a function of the absorbed intensity, with a slight increase of $I_{F}/\left(I_{0}-I_{T}\right)$ from 105 to 127 ± 5. This indicates that the quantum yield has little dependence on the rhodamine concentration. The ratio $I_{F}/\left(I_{0}-I_{T}\right)$ converges to an asymptotic value of 128 ± 5. We associate this optimal ratio to the quantum yield of 0.96 reported for rhodamine 101 in ethanol in the literature [19,20,22].

## 5. Quantum Yield of Nanocrystals

We can now determine the quantum yield of different nanocrystal samples. Given Equation (1) and the previous setup calibration on rhodamine 101 ($I_{F}^{s}/\left(I_{0}^{s}-I_{T}^{s}\right)$ = 128 for $Y^{s}$ = 0.96), we write :

(2) $$Y^{x}=\frac{I_{F}^{x}}{I_{0}^{x}-I_{T}^{x}}\frac{{\left(n^{x}\right)}^{2}}{{\left(n^{s}\right)}^{2}}7.510^{-3}$$

under the condition that the acquisition parameters (time, slit width, detector position...) are the same as for the rhodamine measurements. We estimate the overall relative error to (ΔY/Y)∼10%.

**Figure 4:**
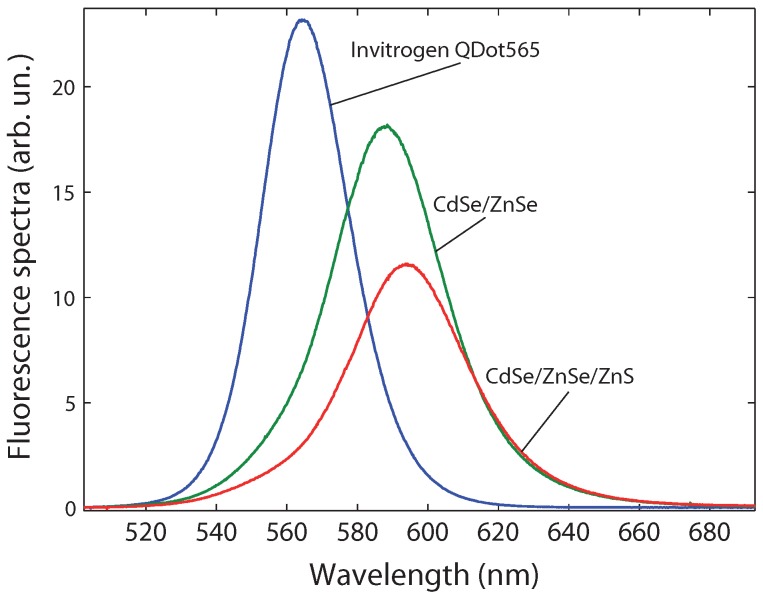
Emission spectra of Invitrogen Qdot565 nanocrystals, and CdSe/ZnSe and CdSe/ZnSe/ZnS nanocrystals.

The emission spectra of the considered samples are plotted in Figure 4. The fluorescence bands range between 500 and 680 nm.

**Figure 5:**
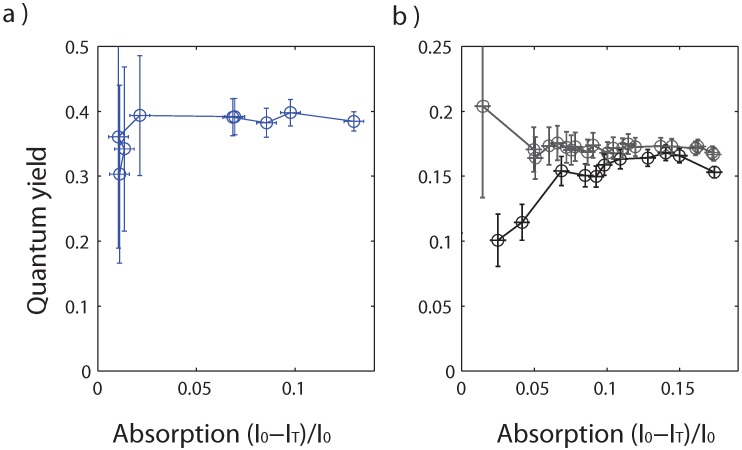
Evolution of the quantum yield of (**a**) Invitrogen Qdot565 and (**b**) CdSe/ZnSe (black) and CdSe/ZnSe/ZnS (grey) nanocrystals as function of the normalized absorption $\left(I_{0}-I_{T}\right)/I_{0}$.

Commercial nanocrystals (Invitrogen QDot565 in decane) were considered as an example of state-of-the-art nanocrystals. Within the experimental margin of error (which can be high for the smaller concentrations), the quantum yield was found to be around 38% for all considered concentrations (Figure 5a).

We now turn to the double-shell CdSe/ZnSe/ZnS nanocrystals synthesized at the Institute of Materials Science (Hanoi). The synthesis protocol has been described in [25,26]. A thick ZnS shell (19 monolayers) is fabricated in order to protect the nanocrystal. Two monolayers of ZnSe are added between CdSe and ZnS as a lattice-parameter matching layer. We use our setup to characterize the quantum yield of an intermediate CdSe/ZnSe sample and of the final CdSe/ZnSe/ZnS nanocrystals, both in toluene (Figure 5b).

We checked that, for CdSe nanocrystals with a thick ZnS shell, the fluorescence intensity was a linear function of the concentration for small concentrations. Above 0.2 *μ*M (CdSe concentration, obtained from the CdSe mass involved in the synthesis reaction), $I_{F}$ was no longer proportional to the concentration. We attribute this to reabsorption of the emitted light, as confirmed by the redshift of the emission peak (4 nm at 0.5 *μ*M), corresponding to a decrease of the high-energy wing of the spectrum. For the following data, we stay at low concentrations in order to avoid reabsorption.

For the intermediate CdSe/ZnSe sample (Figure 5), the quantum yield increases from 10% at lower concentrations to an asymptotic value of 16% at higher concentrations. This illustrates the importance of considering different concentrations. A similar effect was reported in [19] and attributed to the ligand adsorption-desorption equilibrium: at lower concentrations, the ligands are desorbed (as evidenced by other means in [27]) and the nanocrystals are less protected from their environment, leading to a smaller quantum yield.

For the final CdSe/ZnSe/ZnS sample, we find for all concentrations a quantum yield of 17%, with fluctuations of the order of 1%, within the range of experimental error. This is slightly higher than the highest value of 16% for the CdSe/ZnSe sample. Moreover, no decrease of the quantum yield is observed at low concentration. We conclude that the thick ZnS shell provides a better protection than the 2-monolayers ZnSe shell, as there is no effect of ligand desorption on the CdSe/ZnSe/ZnS nanocrystal quantum yield. This study will serve as a guide for future work on improving the nanocrystal photostability and quantum yield.

## 6. Discussion: Quantum Yield and Efficiency

Let us finally compare our present work with a different class of approaches which have used decay measurements to study the quantum yield of nanocrystals [28,29].

The principle of these studies is to change the distance of the nanocrystals to a neighbouring metallic or dielectric interface in order to introduce a controlled change of the photonic density of states. This causes a modification of the nanocrystal radiative decay rate which can be calculated through Fermi’s golden rule. The measured decay curves provide the total (radiative and non-radiative) decay rate of the emitting state. Thus one can obtain the ratio of the radiative rate to the total decay rate of the emitting state, which we call the quantum efficiency of the emitting transition. In [28,29], for nanocrystals similar to our commercial sample, near-unity quantum efficiencies were measured.

The quantum efficiency is, by definition, different from the quantum yield. The quantum yield is the probability that a photon is emitted after one photon has been absorbed [30]. The quantum efficiency is the probability that a photon is emitted after the system has been excited to its emitting state. Nevertheless, in the model of a two-level system, the quantum yield is equal to the emitting state quantum efficiency as each photon absorption excites the system to its emitting state. However, the quantum yield may be lower than the quantum efficiency if more complicated energy levels and decay paths must be taken into account. For instance, a “dark exciton” level is involved at low temperature [31], or biexciton states are achieved at high excitation powers [32].

Let us note also that strong inhomogeneities have been reported within the nanocrystal population.

First, atomic-force microscopy studies, compared with optical single-emitter microscopic observations, have shown that in some cases a large portion of nanocrystals (30% to 50%) can be completely extinct and never emit photons [33].

Second, as explained previously, single-emitter studies show that nanocrystals generally blink from “on” to “off” states. Blinking is not limited to nanocrystals, and has been demonstrated for terrylene [34,35] and green fluorescent protein molecules [36]. Some single-nanocrystal absorption studies have shown that “off” (non-emitting) nanocrystals still absorb light, with an absorption cross-section of the same order as for the “on” state [37].

Measurements of quantum yield such as our present experiment take into account the absorption of both emitting and non-emitting nanocrystals. On the other hand, for the quantum efficiency measurements in [28,29], only “on” nanocrystals contribute to the measured decay rates. The difference between our quantum yield of 38% for state-of-the-art commercial nanocrystals and the reported near-unity quantum efficiencies would suggest that about 60% of our commercial nanocrystals are non-emitting (either momentarily “off” or completely extinct).

Studies of individual nanocrystals are of crucial importance in order to clarify the optical behavior of nanocrystals. Our future efforts will be to obtain more detailed information about the link between individual quantum efficiency measurements and collective scale quantum yields.

## 7. Conclusions

In this paper, we have presented a single-step protocol for the determination of the quantum yield of a fluorophore. In this configuration, the control of the detector position is less critical and the absorption coefficient does not need to be the same for the sample of interest and for the reference dye. Rhodamine 101 has been studied and the results determined particular conditions for its use as a reference dye. Using this protocol, solutions of nanocrystals were analyzed as a function of their absorption. We found a range of concentrations where neither the ligand desorption nor the reabsorption are observed. For our CdSe/ZnSe/ZnS nanocrystals, we find encouraging quantum yields of 17%, which should be improved by better synthesis protocols. We concluded by pointing out the importance of ensemble averages in these experiments, and the necessity of single-nanocrystal experiments.
